# 4497 Accessible False Discovery Rate Computation

**DOI:** 10.1017/cts.2020.164

**Published:** 2020-07-29

**Authors:** Megan C Hollister, Jeffrey D. Blume

**Affiliations:** 1 Vanderbilt University Medical Center

## Abstract

OBJECTIVES/GOALS: To improve the implementation of FDRs in translation research. Current statistical packages are hard to use and fail to adequately convey strong assumptions. We developed a software package that allows the user to decide on assumptions and choose the hey desire. We encourage wider reporting of FDRs for observed findings. METHODS/STUDY POPULATION: We developed a user-friendly R function for computing FDRs from observed p-values. A variety of methods for FDR estimation and for FDR control are included so the user can select the approach most appropriate for their setting. Options include Efron’s Empirical Bayes FDR, Benjamini-Hochberg FDR control for multiple testing, Lindsey’s method for smoothing empirical distributions, estimation of the mixing proportion, and central matching. We illustrate the important difference between estimating the FDR for a particular finding and adjusting a hypothesis test to control the false discovery propensity. RESULTS/ANTICIPATED RESULTS: We performed a comparison of the capabilities of our new p.fdr function to the popular p.adjust function from the base stats-package. Specifically, we examined multiple examples of data coming from different unknown mixture distributions to highlight the null estimation methods p.fdr includes. The base package does not provide the optimal FDR usage nor sufficient estimation options. We also compared the step-up/step-down procedure used in adjusted p-value hypothesis test and discuss when this is inappropriate. The p.adjust function is not able to report raw-adjusted values and this will be shown in the graphical results. DISCUSSION/SIGNIFICANCE OF IMPACT: FDRs reveal the propensity for an observed result to be incorrect. FDRs should accompany observed results to help contextualize the relevance and potential impact of research findings. Our results show that previous methods are not sufficient rich or precise in their calculations. Our new package allows the user to be in control of the null estimation and step-up implementation when reporting FDRs.

